# Elevated High-Density Lipoprotein Cholesterol and Age-Related Macular Degeneration: The Alienor Study

**DOI:** 10.1371/journal.pone.0090973

**Published:** 2014-03-07

**Authors:** Audrey Cougnard-Grégoire, Marie-Noëlle Delyfer, Jean-François Korobelnik, Marie-Bénédicte Rougier, Mélanie Le Goff, Jean-François Dartigues, Pascale Barberger-Gateau, Cécile Delcourt

**Affiliations:** 1 Université de Bordeaux, Bordeaux, France; 2 INSERM (Institut National de la Santé Et de la Recherche Médicale), ISPED (Institut de Santé Publique d’Épidémiologie et de Développement), Centre INSERM U897-Epidemiologie-Biostatistique, Bordeaux, France; 3 Centre Hospitalier Universitaire (CHU) de Bordeaux, Service d’Ophtalmologie, Bordeaux, France; Massachusetts Eye & Ear Infirmary, Harvard Medical School, United States of America

## Abstract

**Background:**

Lipid metabolism and particularly high-density lipoprotein (HDL) may be involved in the pathogenic mechanism of age-related macular degeneration (AMD). However, conflicting results have been reported in the associations of AMD with plasma HDL and other lipids, which may be confounded by the recently reported associations of AMD with HDL-related genes. We explored the association of AMD with plasma lipid levels and lipid-lowering medication use, taking into account most of HDL-related genes associated with AMD.

**Methods:**

The Alienor study is a population-based study on age-related eye diseases performed in 963 elderly residents of Bordeaux (France). AMD was graded from non mydriatic color retinal photographs in three exclusive stages: no AMD (n = 430 subjects, 938 eyes); large soft distinct drusen and/or large soft indistinct drusen and/or reticular drusen and/or pigmentary abnormalities (early AMD, n = 176, 247); late AMD (n = 40, 61). Associations of AMD with plasma lipids (HDL, total cholesterol (TC), Low-density lipoprotein (LDL), and triglycerides (TG)) were estimated using Generalized Estimating Equation logistic regressions. Statistical analyses included 646 subjects with complete data.

**Results:**

After multivariate adjustment for age, sex, educational level, smoking, BMI, lipid-lowering medication use, cardiovascular disease and diabetes, and for all relevant genetic polymorphisms (ApoE2, ApoE4, CFH Y402H, ARMS2 A69S, LIPC rs10468017, LIPC rs493258**,** LPL rs12678919, ABCA1 rs1883025 and CETP rs3764261), higher HDL was significantly associated with an increased risk of early (OR = 2.45, 95%CI: 1.54–3.90; P = 0.0002) and any AMD (OR = 2.29, 95%CI: 1.46–3.59; P = 0.0003). Association with late AMD was far from statistical significance (OR = 1.58, 95%CI: 0.48–5.17; p = 0.45). No associations were found for any stage of AMD with TC, LDL and TG levels, statin or fibrate drug use.

**Conclusions:**

This study suggests that elderly patients with high HDL concentration may be at increased risk for AMD and, further, that HDL dysfunction might be implicated in AMD pathogenesis.

## Introduction

Age-related macular degeneration (AMD) is the leading cause of blindness in high-income countries, and the third global cause of blindness in the world [1]. This disease affects 2.5 million subjects in Europe [2] and 1.75 million in the USA [3]. While the pathophysiology of AMD remains elusive, a number of risk factors, such as smoking, nutrition and several genetic polymorphisms have been evidenced [4]. The early stage of AMD is characterized by the presence of large and soft drusen (extracellular deposits, seen as yellow spots on the retina) and/or of pigmentary abnormalities. The later stages involve atrophy of the retinal pigment epithelium (RPE) (dry form) or the development of choroidal neovascularization (wet form).

With advancing age, there is a deposit of lipid particles in normal Bruch’s membrane (BrM) leading to the creation of a lipid wall, external to the RPE basal lamina, impairing nutrient exchange between the choriocapillaris and the RPE and compromising retinal function [5]–[8]. The observation that the location of the lipid wall was the same as and precedes the basal linear deposits and drusen suggested its contribution to drusen formation [6], [8]. Indeed, lipids (both esterified and unesterified cholesterol, and phosphatidylcholine) represent at least 40% of the volume of drusen [6], [9], [10].

Furthermore, within the genomic era, several lipid-related genes have been reported to be associated with AMD. The E4 allele of the Apolipoprotein E gene (ApoE4) is associated with a reduced risk for AMD, while the ApoE2 increases the risk for AMD [11], [12]. ApoE plays a key role in cholesterol metabolism [13] and is associated with macular pigment optical density [14]. Other genes present particularly in the high-density lipoprotein cholesterol (HDL) pathway as the hepatic lipase gene (LIPC) [15]–[22], the lipoprotein lipase gene (LPL) [15], [17], [18], the cholesterol ester transferase gene (CETP) [16], [17], [21] and the ABC-binding cassette A1 (ABCA1) gene [15]–[18], [21] have been shown to be associated with AMD in genome-wide association (GWAS) studies and have been confirmed in several epidemiological studies [21], [23], including the Alienor study [22].

These findings have led to the hypothesis that lipid metabolism and particularly HDL is involved in the pathogenic mechanism of AMD [6], [9], [24]. However, conflicting results have been reported with regard to the associations of AMD with serum HDL concentration. An increasing number of studies reported higher risk for AMD among subjects with elevated HDL [25]–[31], few others reported a reduced risk [32]–[34] and some others reported no significant associations [35]–[38].

Other associations between serum lipids such as total cholesterol (TC), low-density lipoprotein (LDL) and triglycerides (TG), and AMD have also been investigated with still conflicting findings. In 1992, the Eye Disease Case Control Study Group, reported a significant 4-fold increased risk for exudative AMD with the highest serum cholesterol concentration [39]. Since then, only five studies reported a significant increased risk of AMD with high level of total cholesterol [23], [33], [40]–[42], while 13 reported no significant association [26], [27], [30], [32], [34]–[38], [43]–[46], and two an inverse relationship [47], [48].

Recently, Ebrahimi *et al* suggested that before excluding the role of systemic lipids in AMD, the role of plasma lipids in the context of genotype could be examined to identify predisposition in a subset of patients at risk for AMD due to genotype and plasma lipid levels [24].

Thus, the aim of this study was to explore the association between plasma lipid levels, lipid-lowering drug use and AMD, taking into account most of HDL-related genes associated with AMD, in the framework of a population-based study.

## Subjects and Methods

### Study Purpose

The Alienor (Antioxydants, LIpids Essentiels, Nutrition et maladies OculaiRes) Study is a population-based study aimed at assessing the associations of age-related eye diseases (AMD, glaucoma, cataract, dry eye syndrome) with nutritional factors (in particular antioxidants, macular pigment, and fatty acids), determined from plasma measurements and estimations of dietary intake [49]. It also takes into account other major determinants of eye disease, including gene polymorphisms, lifestyle and vascular factors.

### Study Sample

**Figure 1:**
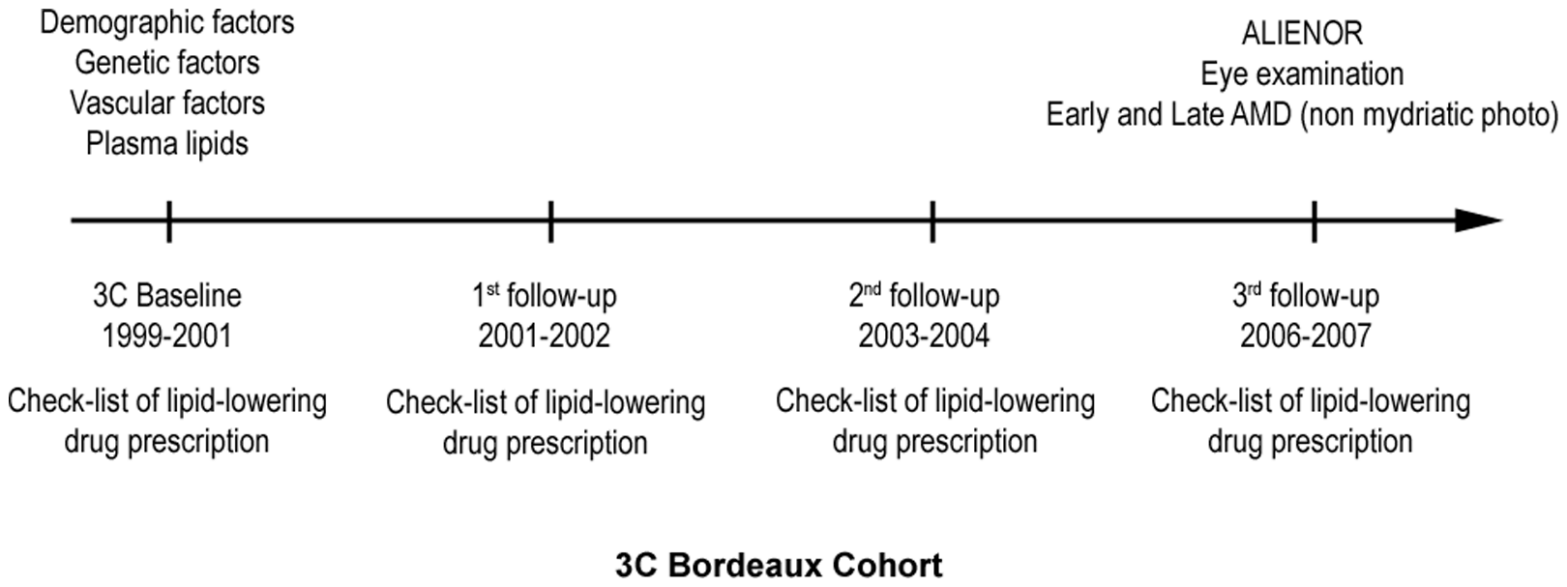
Design of the Alienor study. Abbreviations: AMD: age-related macular degeneration.

Subjects of the Alienor Study were recruited from an ongoing population-based study (Three-City [3C] Study) on the vascular risk factors for dementia [50]. The 3C Study included 9294 subjects aged 65 years and older from three French cities (Bordeaux, Dijon, Montpellier), among whom 2104 were recruited in Bordeaux. Subjects were contacted individually from the electoral rolls. They were initially recruited in 1999 to 2001 and were followed-up about every 2 years since baseline (Figure 1). Data collected at each examination included cognitive testing with diagnoses of dementia and assessment of vascular risk factors. In addition, fasting blood and DNA samples were collected at baseline and kept frozen at −80°C.

The Alienor Study consisted in an eye examination, which was proposed to all participants of the third follow-up (2006–2008) of the 3C cohort in Bordeaux. Among the 1450 alive participants re-examined in 2006 to 2008, 963 (66.4%) participated in the Alienor Study, and 487 (33.6%) declined participation. Detailed characteristics of participants and nonparticipants have been published elsewhere [49].

This research followed the tenets of the Declaration of Helsinki. Participants gave written consent for participation in the study. The design of the Alienor Study has been approved by the Ethical Committee of Bordeaux (Comite de Protection des Personnes Sud-Ouest et Outre-Mer III) in May 2006.

### Eye Examination

The eye examination took place in the Department of Ophthalmology of the University Hospital of Bordeaux, France. It included a recording of ophthalmological history, measures of visual acuity, refraction, two 45° nonmydriatic color retinal photographs (one centered on the macula, the other centered on the optic disc), measures of intraocular pressure and central corneal thickness, and break-up time test. A self-completed questionnaire on risk factors specific to the eye and dry eye symptoms was completed at home and brought back on the day of the eye examination.

Retinal photographs were performed using a nonmydriatic retinograph (TRC NW6S; Topcon, Tokyo, Japan) and were interpreted in duplicate by two specially trained technicians. Inconsistencies between the two interpretations were adjudicated by a retina specialist for classification of AMD and other retinal diseases and by a glaucoma specialist for classification of glaucoma. All cases of late AMD, other retinal diseases, and glaucoma were reviewed and confirmed by specialists.

### Classification of AMD

Retinal photographs were interpreted according to the international classification [51] and to a modification of the grading scheme used in the Multi-Ethnic Study of Atherosclerosis for drusen size, location, and area [52]. Late AMD was defined by the presence of neovascular AMD or geographic atrophy within the grid (3000 µm from the foveola). Neovascular AMD included serous or hemorrhagic detachment of the retinal pigment epithelium (RPE) or sensory retina, subretinal or sub-RPE hemorrhages, and fibrous scar tissue. Geographic atrophy was defined as a discrete area of retinal depigmentation, 175 µm in diameter or larger, characterized by a sharp border and the presence of visible choroidal vessels. Five cases of late AMD had no gradable photographs and were classified by ophthalmological history of AMD and AMD therapy (in particular, antiangiogenic agents and photodynamic therapy) and confirmed by their treating ophthalmologist. Early AMD was defined by the presence of soft distinct drusen and/or soft indistinct drusen and/or reticular drusen and/or pigmentary abnormalities. Soft distinct and soft indistinct drusen were larger than 125 µm in diameter and had, respectively, uniform density and sharp edges or decreasing density from the center outward and fuzzy edges. Pigmentary abnormalities were defined as areas of hyperpigmentation and/or hypopigmentation (without visibility of choroidal vessels). Eyes were classified according to 3 exclusive groups: no AMD, early AMD, late AMD.

### Plasma Lipids Measurements

Plasma measurements were determined from fasting blood samples collected at the 3C baseline visit (1999–2001) into heparinized evacuated tubes and centrifuged at 1000 g for 10 min. Plasma lipids (TC, LDL, HDL, and TG) were measured at the Biochemistry Laboratory of the University Hospital of Dijon.

### Lipid Lowering Medications

The clinical baseline (from 1999 to 2001) and follow-up examinations (1^st^ follow-up examination from 2001 to 2002; 2^nd^ follow-up examination from 2003 to 2004 and 3^rd^ follow-up examination from 2006 to 2007) included an inventory of all drugs used during the preceding month. Medical prescriptions and, where feasible, the medications themselves were seen by the interviewer. The name of the medication was recorded, and all drugs were subsequently coded according to the French translation of the world health organization (WHO) anatomical therapeutic chemical (ATC) classification [53]. Three classes of lipid-lowering medications were defined according to the ATC classification: 1) Statins (ATC codes: C10AA; C10BA; C10BX); 2) Fibrates (ATC codes: C10AB); 3) Others (ATC codes: C10AC; C10AD; C10AX). Lipid-lowering medication use was defined as the use of at least one of the preceding classes of drugs between the baseline and the last follow-up.

### Other Variables

The following potential confounders have been selected based on literature results reporting significant associations of AMD or serum lipid concentrations with age, gender, educational level, smoking, body mass index (BMI), hypertension, cardiovascular disease, diabetes, Complement Factor H (CFH) Y402H (rs1061170), Age-Related Maculopathy Susceptibility 2 (ARMS2, rs10490924) A69S, apolipoproteins E2 (ApoE2) and E4 (ApoE4), LIPC (rs10468017), LIPC (rs493258), LPL (rs12678919), ABCA1 (rs1883025) and CETP (rs3764261) polymorphisms.

Data were collected during a face-to-face interview using a standardized questionnaire administered by a trained psychologist or nurse. At baseline, general data included: demographic characteristics, educational level and smoking. BMI (kg/m^2^) was calculated as weight/height^2^ using weight and height measured at baseline. Two separate measures of blood pressure in a seated position were performed in all participants. The first blood pressure measurement was recorded at the beginning of the interview and the second one at the end, using a digital electronic tensiometer (OMRON M4, France). The average systolic blood pressure (SBP) was the average of these two SBP measures. The same calculation was made for the average diastolic blood pressure (DBP). Hypertension was defined as average SBP≥140 mmHg and/or average DBP≥90 mmHg and/or antihypertensive medication use at baseline examination.

Genetic polymorphisms were determined by the Lille Génopôle, from the DNA samples collected at baseline (1999–2001). The included genetic factors have been shown to be very strong predictors of risk for AMD and/or cardiovascular disease in previous studies, including the Alienor Study [19], [54]–[56].

### Statistical Analyses

Associations of baseline demographic, behavioural, anthropometric, medical and genetic characteristics with plasma lipid concentrations, statin and fibrate drug use were examined with Student test, Analysis of variance (ANOVA), and Chi-square test, as appropriate.

Associations of early and late AMD with plasma lipid variables, statin and fibrate drug use were estimated using logistic Generalized Estimating Equation (GEE) models, taking into account the data from both eyes and their intra-individual correlation. [57] In all analyses, subjects without any AMD were considered as the reference group.

GEE models for plasma lipids, statin and fibrate drug use were adjusted first for age and gender only (Model 1); in Model 2 we performed additional adjustment for educational level (no education or primary school or short secondary school *vs.* long secondary school or high school or university), smoking (never, 1 to less than 20 pack-years, 20 pack-years or more), BMI (kg/m^2^, <25, [25]–[30], ≥30), hypertension, cardiovascular disease, diabetes (Fasting glycemia ≥6.1 mmol/L or nonfasting glycemia ≥11.0 mmol/l or antidiabetic medication) and for lipid-lowering medications (at one examination or more), for plasma lipid concentrations; and for HDL, LDL and TG, only for statin and fibrate drug use. Finally, Model 3 was adjusted with all previous variables and genetic risk factors (ApoE2, ApoE4, CFH Y402H, ARMS2 A69S, LIPC rs10468017, LIPC rs493258, LPL rs12678919, ABCA1 rs1883025 and CETP rs3764261 polymorphisms).

Potential interactions between plasma lipid levels and lipid-lowering medication use and between plasma lipid levels and genetic polymorphisms were assessed. Lipid-lowering medications use and genetic polymorphisms were introduced in the models one by one. We withdrew interaction terms when not statistically significant (*P*>0.05).

False positive results are a critical concern when a number of association tests are performed. To address this issue, we applied Bonferroni correction to correct for multiple testing.

All statistical analyses were performed using SAS version 9.2 (SAS Institute Inc, Cary, NC; procedure GENMOD for the GEE analysis).

## Results

Among the 963 subjects of the ALIENOR study, subjects were aged 80.2 years on average and the proportion of women was 61.9%. Among the 963 subjects, 84 (8.7%) subjects had ungradable photographs in both eyes and 54 (5.6%) had missing data for plasma lipid measurement and/or lipid-lowering drug use. Thus the statistical analyses were conducted on 825 subjects (85.7%) corresponding to 1595 eyes.

Table 1 presents the plasma lipid concentrations and lipid lowering drug use of the ALIENOR subjects according to AMD status. HDL was higher in early and late AMD cases, while there were no differences for TC, LDL or TG. No associations were found for use of statin or fibrates.

**Table 1:** Plasma lipid levels and statin and fibrate drug use according to AMD status, in subjects of the Alienor study (N = 825).

	TC (mM)	LDL (mM)	HDL (mM)	TG (mM)	Statin*			Fibrates†		
					No (N = 507)	Yes (N = 318)		No (N = 663)	Yes (N = 162)	
	Mean (SD)	Mean (SD)	Mean (SD)	Mean (SD)	N (%)		N (%)	N (%)		N (%)
**AMD**										
None (N = 540)	5.78 (0.95)	3.66 (0.83)	1.56 (0.38)	1.24 (0.59)	331 (65.3)	209 (65.72)		441 (66.52)	99 (61.11)	
Early AMD (N = 238)	5.81 (0.98)	3.62 (0.85)	1.66 (0.41)	1.17 (0.51)	145 (28.6)	93 (29.25)		183 (27.60)	55 (33.95)	
Late AMD (N = 47)	5.72 (1.17)	3.48 (0.96)	1.69 (0.45)	1.21 (0.66)	31 (6.1)	16 (5.03)		39 (5.88)	8 (4.94)	
*P value* ‡	*P = 0.83*	*P = 0.36*	*P = 0.0009*	*P = 0.26*	*P = 0.80*			*P = 0.27*		

Abbreviations: AMD: age-related macular degeneration; TC: Total cholesterol; TG: Triglycerides;

*Use of statin at one examination or more;

† Use of fibrate at one examination or more;

‡ ANOVA were performed to compare the difference in distribution of plasma lipid levels between AMD stages; χ^2^ tests were performed to compare the differences in statin and fibrates drug uses between AMD stages.

Tables 2 and 3 present the associations of plasma lipids and lipid lowering medications with genetic polymorphisms. After Bonferroni correction (P = 0.0055), subjects with at least one Apo E2 allele had lower mean level of TC and LDL and higher mean level of TG than those with no Apo E2 allele. Subjects with at least one Apo E4 allele used significantly more statins than subjects with no ApoE4 allele. For CETP rs3764261, subjects with AA genotype had significantly higher mean level of HDL than subjects with CC or AC genotypes. No associations were found between ApoE4, CFH Y402H, ARMS2 A69S, LIPC rs493258, LIPC rs10468017, ABCA1 rs1883025 polymorphisms and plasma lipid levels. No associations were found between CFH Y402H, ARMS2 A69S, LIPC rs493258, LIPC rs10468017, LPL, ABCA1 and CETP polymorphisms and statin and fibrate drug use.

**Table 2:** Plasma lipid levels according to genetic characteristics in subjects of the Alienor study (N = 825).

	TC (mM)	LDL (mM)	HDL (mM)	TG (mM)
	Mean (SD)	Mean (SD)	Mean (SD)	Mean (SD)
**ApoE2 (N = 815)**				
No allele E2 (N = 711)	5.83 (1.00)	3.70 (0.86)	1.58 (0.39)	1.20 (0.53)
At least 1 allele E2 (N = 104)	5.53 (0.78)	3.26 (0.66)	1.64 (0.40)	1.37 (0.77)
*P value* * ^,^ †	*0.004*	*<0.0001*	*0.15*	*0.004*
**ApoE4 (N = 815)**				
No allele E4 (N = 670)	5.78 (0.98)	3.62 (0.85)	1.60 (0.39)	1.22 (0.57)
At least 1 allele E4 (N = 145)	5.84 (0.96)	3.71 (0.83)	1.58 (0.39)	1.20 (0.58)
*P value* * ^,^ †	*0.48*	*0.23*	*0.54*	*0.72*
**LPL rs12678919 (N = 714)**				
A A (N = 531)	5.76 (0.97)	3.62 (0.86)	1.57 (0.39)	1.25 (0.59)
A G (N = 170)	5.80 (0.97)	3.70 (0.81)	1.57 (0.37)	1.16 (0.49)
G G (N = 13)	5.37 (0.80)	3.10 (0.63)	1.83 (0.43)	0.97 (0.51)
*P value* * ^,^ †	*0.32*	*0.04*	*0.06*	*0.04*
**CETP rs3764261 (N = 719)**				
C C (N = 377)	5.74 (0.97)	3.66 (0.86)	1.51 (0.34)	1.26 (0.61)
A C (N = 287)	5.77 (0.99)	3.59 (0.84)	1.63 (0.41)	1.20 (0.53)
A A (N = 55)	5.90 (0.93)	3.59 (0.78)	1.81 (0.43)	1.12 (0.43)
*P value* * ^,^ †	*0.50*	*0.60*	*<0.0001*	*0.14*
**LIPC rs493258 (N = 736)**				
C C (N = 215)	5.79 (0.99)	3.65 (0.86)	1.56 (0.39)	1.28 (0.63)
T C (N = 352)	5.76 (0.94)	3.63 (0.84)	1.58 (0.37)	1.21 (0.53)
T T (N = 169)	5.76 (1.03)	3.60 (0.84)	1.61 (0.41)	1.22 (0.58)
*P value* * ^,^ †	*0.95*	*0.84*	*0.43*	*0.39*
**LIPC rs10468017 (N = 723)**				
C C (N = 375)	5.75 (0.98)	3.64 (0.87)	1.57 (0.38)	1.21 (0.57)
T C (N = 289)	5.77 (0.96)	3.61 (0.83)	1.60 (0.38)	1.23 (0.52)
T T (N = 59)	5.76 (1.07)	3.57 (0.88)	1.61 (0.45)	1.28 (0.73)
*P value* * ^,^ †	*0.98*	*0.81*	*0.50*	*0.64*
**ABCA1 rs1883025 (N = 736)**				
C C (N = 390)	5.79 (1.02)	3.63 (0.87)	1.61 (0.39)	1.22 (0.53)
C T (N = 290)	5.75 (0.93)	3.62 (0.83)	1.56 (0.38)	1.24 (0.62)
T T (N = 57)	5.70 (0.87)	3.63 (0.74)	1.49 (0.37)	1.28 (0.62)
*P value* * ^,^ †	*0.70*	*0.98*	*0.07*	*0.73*
**CFH Y402H (N = 802)**				
T T (N = 362)	5.74 (0.93)	3.60 (0.81)	1.59 (0.39)	1.21 (0.56)
T C (N = 346)	5.82 (0.99)	3.67 (0.85)	1.61 (0.41)	1.19 (0.52)
C C (N = 94)	5.82 (1.11)	3.63 (0.99)	1.59 (0.38)	1.31 (0.65)
*P value* * ^,^ †	*0.51*	*0.53*	*0.81*	*0.17*
**ARMS2 A69S (N = 736)**				
G G (N = 475)	5.79 (0.96)	3.60 (0.81)	1.59 (0.39)	1.21 (0.56)
G T (N = 234)	5.75 (0.98)	3.67 (0.85)	1.61 (0.41)	1.19 (0.52)
T T (N = 27)	5.56 (1.18)	3.63 (0.99)	1.59 (0.38)	1.31 (0.65)
*P value* * ^,^ †	*0.46*	*0.53*	*0.81*	*0.17*

Abbreviations: TC: Total cholesterol; TG: Triglycerides;

*ANOVA or Student t-tests were performed for means comparison;

† Statistically significant p-value <0.0055 (Bonferroni corrected, P = 0.05/9 = 0.0055).

**Table 3:** Statin and fibrate drug use according to genetic characteristics in subjects of the Alienor study (N = 825).

	Statin*		Fibrates†	
	No (N = 507)	Yes (N = 318)	No (N = 663)	Yes (N = 162)
	N (%)	N (%)	N (%)	N (%)
**ApoE2 (N = 815)**	(N = 500)	(N = 315)	(N = 656)	(N = 159)
No allele E2 (N = 711)	427 (85.4)	284 (90.2)	569 (86.7)	142 (89.3)
At least 1 allele E2 (N = 104)	73 (14.6)	31 (9.8)	87 (13.3)	17 (10.7)
*P value* ‡ ^,^ §	*0.05*		*0.38*	
**ApoE4 (N = 815)**	(N = 500)	(N = 315)	(N = 656)	(N = 159)
No allele E4 (N = 670)	432 (86.4)	238 (75.6)	544 (82.9)	126 (79.2)
At least 1 allele E4 (N = 145)	68 (13.6)	77 (24.4)	112 (17.1)	33 (20.8)
*P value* ‡ ^,^ §	*<0.0001*		*0.28*	
**LPL rs12678919 (N = 714)**	(N = 437)	(N = 277)	(N = 580)	(N = 134)
A A (N = 531)	323 (73.9)	208 (75.1)	430 (74.1)	101 (75.4)
A G (N = 170)	105 (24.0)	65 (23.5)	139 (24.0)	31 (23.1)
G G (N = 13)	9 (2.1)	4 (1.4)	11 (1.9)	2 (1.5)
*P value* ‡ ^,^ §	*0.82*		*0.93*	
**CETP rs3764261 (N = 719)**	(N = 440)	(N = 279)	(N = 585)	(N = 134)
C C (N = 377)	225 (51.1)	152 (54.5)	309 (52.8)	68 (50.8)
A C (N = 287)	177 (40.2)	110 (39.4)	228 (39.0)	59 (44.0)
A A (N = 55)	38 (8.6)	17 (6.1)	48 (8.2)	7 (5.2)
*P value* ‡ ^,^ §	*0.40*		*0.36*	
**LIPC rs493258 (N = 736)**	(N = 452)	(N = 284)	(N = 599)	(N = 137)
C C (N = 215)	130 (28.8)	85 (29.9)	184 (30.7)	31 (22.6)
T C (N = 352)	217 (48.0)	135 (47.5)	280 (46.7)	72 (52.5)
T T (N = 169)	105 (23.2)	64 (22.5)	135 (22.5)	34 (24.8)
*P value* ‡ ^,^ §	*0.94*		*0.17*	
**LIPC rs10468017 (N = 723)**	(N = 443)	(N = 280)	(N = 588)	(N = 135)
C C (N = 375)	230 (51.9)	145 (51.8)	311 (52.9)	64 (47.4)
T C (N = 289)	175 (39.5)	114 (40.7)	228 (38.8)	61 (45.2)
T T (N = 59)	38 (8.6)	21 (7.5)	49 (8.3)	10 (7.4)
*P value* ‡ ^,^ §	*0.86*		*0.39*	
**ABCA1 rs1883025 (N = 736)**	(N = 452)	(N = 284)	(N = 599)	(N = 137)
C C (N = 390)	247 (54.6)	143 (50.3)	315 (52.6)	75 (54.8)
C T (N = 290)	173 (38.3)	116 (40.8)	238 (39.7)	51 (37.2)
T T (N = 57)	32 (7.1)	25 (8.8)	46 (7.7)	11 (8.0)
*P value* ‡ ^,^ §	*0.46*		*0.86*	
**CFH Y402H (N = 802)**	(N = 494)	(N = 308)	(N = 643)	(N = 159)
T T (N = 362)	226 (45.7)	136 (44.2)	284 (44.2)	78 (49.1)
T C (N = 346)	218 (41.6)	128 (41.6)	281 (43.7)	65 (40.9)
C C (N = 94)	50 (14.3)	44 (14.3)	78 (12.1)	16 (10.1)
*P value* ‡ ^,^ §	*0.20*		*0.50*	
**ARMS2 A69S (N = 736)**	(N = 452)	(N = 284)	(N = 599)	(N = 117)
G G (N = 475)	291 (64.4)	184 (64.8)	390 (65.1)	85 (62.0)
G T (N = 234)	141 (31.2)	93 (32.7)	185 (30.9)	49 (35.8)
T T (N = 27)	20 (4.4)	7 (2.5)	24 (4.0)	3 (2.2)
*P value* ‡ ^,^ §	*0.37*		*0.37*	

* Use of statin at one examination or more;

† Use of fibrate at one examination or more;

‡ χ^2^ tests were performed for frequency comparison;

§ Statistically significant p-value <0.0055 (Bonferroni corrected, P = 0.05/9 = 0.0055).

Associations with baseline demographic, behavioural and anthropometric characteristics and plasma lipid concentrations are presented in Table S1 in File S1. After Bonferroni correction (P = 0.0083), TC and HDL were significantly higher in women, never smokers and subjects with lower BMI. TG was significantly higher in smokers and subjects with higher BMI. No associations were found for LDL, Statin and Fibrates use (Table S1 in File S1). With regard to medical characteristics at baseline, after Bonferroni correction (P<0.017), TC and LDL were significantly higher in the absence of cardiovascular disease, HDL was higher in the absence of cardiovascular disease, hypertension or diabetes, and TG was higher in the presence of cardiovascular disease, hypertension or diabetes (Table S2 in File S1). Statin use was significantly associated with the presence of cardiovascular disease, hypertension and diabetes, and having higher levels of LDL and TG and lower level of HDL. No such associations were found for use of fibrates (Tables S2 and S3 in File S1).

Table 4 presents association of AMD with plasma lipids levels, statin and fibrate drug use. After adjustment for age and sex (model 1) and Bonferroni correction (P = 0.0125), higher HDL was significantly associated with an increased risk of early and any AMD (OR ranging from 1.77–1.84 per 1 mmol/L, P values ranging from 0.0008–0.003). HDL was associated with late AMD (OR = 2.41, P = 0.04) with a P value<0.05, but did not remained significant after Bonferroni correction. No associations were found for any stage of AMD with TC, LDL and TG concentrations, or statin and fibrate drug use.

**Table 4:** Associations of AMD with plasma lipids levels in the Alienor study (odds-ratios (OR) and [95% confidence interval (CI)] for 1 mmol/L increase).

			Model 1* (N = 1595 eyes)			Model 2† (N = 1518 eyes)			Model 3‡ (N = 1246 eyes)		
		None	Any AMD	Early AMD	Late AMD	Any AMD	Early AMD	Late AMD	Any AMD	Early AMD	Late AMD
			(n = 410)	(n = 336)	(n = 74)	(n = 376)	(n = 307)	(n = 69)	(n = 308)	(n = 247)	(n = 61)
**TC**		ref	1.02	1.03	1.00	0.99	0.99	1.01	1.03	1.03	0.99
			[0.88–1.17]	[0.89–1.19]	[0.69–1.46]	[0.86–1.15]	[0.85–1.15]	[0.69–1.50]	[0.88–1.21]	[0.88–1.21]	[0.68–1.45]
	*P value* §		*0.81*	*0.71*	*0.98*	*0.93*	*0.88*	*0.94*	*0.69*	*0.70*	*0.97*
**LDL**		ref	0.93	0.96	0.86	0.91	0.92	0.86	0.93	0.93	0.88
			[0.79–1.10]	[0.81–1.13]	[0.57–1.30]	[0.77–1.08]	[0.77–1.10]	[0.55–1.36]	[0.78–1.12]	[0.78–1.12]	[0.59–1.32]
	*P value* §		*0.40*	*0.60*	*0.47*	*0.28*	*0.36*	*0.53*	*0.47*	*0.47*	*0.55*
**HDL**		ref	1.84	1.77	2.41	1.83	1.71	2.58	2.29	2.45	1.58
			[1.29–2.63]	[1.22–2.57]	[1.03–5.64]	[1.25–2.70]	[1.15–2.54]	[0.95–7.05]	[1.46–3.59]	[1.54–3.90]	[0.48–5.17]
	*P value* §		*0.0008*	*0.003*	*0.04*	*0.002*	*0.008*	*0.06*	*0.0003*	*0.0002*	*0.45*
**TG**		ref	0.87	0.85	0.92	0.86	0.83	0.96	0.85	0.79	1.30
			[0.67–1.13]	[0.65–1.11]	[0.49–1.71]	[0.64–1.14]	[0.62–1.11]	[0.47–1.97]	[0.62–1.17]	[0.56–1.12]	[0.48–3.52]
	*P value* §		*0.30*	*0.24*	*0.78*	*0.28*	*0.21*	*0.90*	*0.33*	*0.19*	*0.61*

Abbreviations: AMD: age-related macular degeneration; TC: Total cholesterol; TG: Triglycerides;

*adjusted for age and gender;

† adjusted for age, gender, educational level, smoking, BMI, hypertension, hypolipidemic drug, cardiovascular disease, and diabetes;

‡ adjusted for age, gender, educational level, smoking, BMI, hypertension, hypolipidemic drug, cardiovascular disease, diabetes, ApoE2, ApoE4, CFH Y402H, ARMS2 A69S, LIPC(rs10468017), LIPC(rs493258) LPL, ABCA1 and CETP polymorphisms;

§ Statistically significant p-value <0.0125 (Bonferroni corrected, P = 0.05/4 = 0.0125).

After further adjustment for educational level, smoking, BMI, lipid-lowering medication use, cardiovascular disease and diabetes, higher HDL remained significantly associated with an increased risk of early (OR = 1.71, P = 0.008) or any AMD (OR = 1.83, P = 0.002) and tended to be associated with increased risk of late AMD, but did not reach statistical significance (OR = 2.58, P = 0.06) especially after Bonferroni correction. No associations were found for any stage of AMD with TC, LDL and TG levels and statin and fibrate drug use.

After further adjustment for all relevant genetic polymorphisms (apoE2, ApoE4, CFH Y402H, ARMS2 A69S, LIPC rs10468017, LIPC rs493258**,** LPL rs12678919, ABCA1 rs1883025 and CETP rs3764261), higher HDL remained significantly associated with an increased risk of early (OR = 2.45, P = 0.0002) or any AMD (OR = 2.29, P = 0.0003). Association with late AMD was far from statistical significance (OR = 1.58, p = 0.45). No associations were found for any stage of AMD with TC, LDL and TG levels and statin and fibrate drug use (Table 5).

**Table 5:** Associations of AMD with statin and fibrate drug use in the Alienor study (odds-ratios (OR) and [95% confidence interval (CI)] for 1 mmol/L increase).

			Model 1* (N = 1595 eyes)			Model 2† (N = 1518 eyes)			Model 3‡ (N = 1246 eyes)		
		None	Any AMD	Early AMD	Late AMD	Any AMD	Early AMD	Late AMD	Any AMD	Early AMD	Late AMD
			(n = 410)	(n = 336)	(n = 74)	(n = 376)	(n = 307)	(n = 69)	(n = 308)	(n = 247)	(n = 61)
**Statin use** ∥		ref	0.99	1.01	0.78	1.05	1.10	0.68	1.16	1.22	0.72
			[0.75–1.32]	[0.75–1.36]	[0.41–1.49]	[0.77–1.44]	[0.79–1.53]	[0.33–1.38]	[0.80–1.70]	[0.82–1.81]	[0.31–1.70]
	*P value* #		*0.96*	*0.95*	*0.45*	*0.75*	*0.57*	*0.29*	*0.43*	*0.33*	*0.46*
**Fibrates use** β		ref	1.24	1.32	0.97	1.26	1.36	0.95	1.44	1.50	1.32
			[0.89–1.73]	[0.93–1.86]	[0.43–2.15]	[0.89–1.80]	[0.94–1.96]	[0.41–2.24]	[0.96–2.17]	[0.98–2.29]	[0.50–3.52]
	*P value* #		*0.20*	*0.11*	*0.93*	*0.20*	*0.10*	*0.92*	*0.08*	*0.06*	*0.58*

Abbreviations: AMD: age-related macular degeneration;

*adjusted for age and gender;

† adjusted for age, gender, educational level, smoking, BMI, hypertension, HDL, LDL, triglycerides, cardiovascular disease, and diabetes;

‡ adjusted for age, gender, educational level, smoking, BMI, hypertension, HDL, LDL, triglycerides, cardiovascular disease, diabetes, ApoE2, ApoE4, CFH Y402H, ARMS2 A69S, LIPC(rs10468017**)**, LIPC(rs493258**)** LPL, ABCA1 and CETP polymorphisms;

∥ Use of statin at one examination or more;

# Statistically significant p-value <0.025 (Bonferroni corrected, P = 0.05/2 = 0.025);

β Use of fibrate at one examination or more.

No significant interactions were found between plasma lipid levels and genetic polymorphisms, or between plasma lipid levels and lipid-lowering medication use, with regard to the risk for AMD, suggesting that genetic background and use of lipid-lowering medication do not modify the association of plasma lipid levels with AMD (data not shown).

## Discussion

In the present study, after full adjustment, elevated HDL was significantly associated with an increased risk of any and early AMD, independent of many potential confounders, including the major genetic polymorphisms involved in the risk for AMD. Results for late AMD were in the same direction, but far from statistical significance. No associations were found for any stage of AMD with other plasma lipids as well as statin or fibrate drug use.

Our results are consistent with findings of several previous studies which did not adjust for genetic polymorphisms. In cross-sectional studies, high HDL concentration was associated with early AMD in the Beaver Dam study [28]; with soft drusen in the POLA study [26] and with AMD in the Oklahoma Indians population AMD [25]. In a case-control study, Hyman *et al* reported a positive association between HDL and neovascular AMD (OR of highest quintile vs. lowest quintile serum HDL, 2.3; 95%CI, 1.1–4.7) [27]. In the prospective Rotterdam study, HDL was associated with an increased incidence of any AMD [31]. In the Beaver Dam study, higher HDL at baseline was associated with the 10 year incidence of geographic atrophy (RR per 10 mg/dl HDL cholesterol, 1.29; 95% CI, 1.05–1.58; P = 0.01) [30].

Data from other studies have been inconsistent regarding the association between HDL and AMD. In the Blue Mountains Eye Study, there was no significant cross-sectional association of HDL with geographic atrophy (OR: 0.82; 95%CI: 0.19–3.46), or exudative macular degeneration (OR: 1.73; 95%CI: 0.83–3.62) [37], whereas elevated HDL was associated with a decreased 5-years incidence of late AMD (RR per standard deviation increase, 0.74; 95% CI, 0.56–0.99) [34]. In the Beaver Dam study Offspring study, higher HDL was associated with lower risk of early AMD (OR per 5 mg.dl:0.91, 95%CI: 0.83–0.998) [32]. In a case control study, Nowak *et al* found a significant decrease of HDL concentration in AMD patients in comparison with controls, supported by the findings of Reynolds *et al*. where elevated HDL was associated with a reduced risk of late AMD (P<0.05), especially for the neovascular form (P = 0.03) [23], [33]. The pooled data from the Beaver Dam, the Blue Mountains and the Rotterdam studies showed no significant associations between HDL and incident AMD [38]. The cross sectional study of the Singapore Malay Eye Study (SiMES) found no significant associations between HDL and early (OR per mmol/l: 1.14; 95%CI: 0.72–1.82) or late AMD (OR per mmol/l: 0.42; 95%CI: 0.10–1.80) [35]. Similarly, a meta-analysis did not report significant associations between HDL and late AMD for prospective cohort studies (RR: 1.00; 95%CI: 0.97–1.02) as well as cross sectional studies (OR: 1.06; 95%CI: 0.80–1.39) or case-control study (RR: 3.35; 95%CI: 0.92–12.23) [36].

In the present study, no statistically significant associations of AMD with TC, LDL or TG were found. Findings on cholesterol have been inconsistent in the literature. Some studies reported that elevated total cholesterol concentration was associated with an increased risk of AMD [23], [33], [39]–[42]. In contrast, few studies found a significant inverse relation between total serum cholesterol and AMD [47], [48] while numerous other studies reported no significant association [26], [27], [30], [32], [35], [36], [43]–[45]. Few studies reported an increased risk of AMD with high level of LDL [23], [33], [38], [58] and TG [58].

We found no association between statin or fibrate drug use and AMD. In the literature, the association between the use of cholesterol-lowering medications and AMD has been intensively studied. Again, the results have been inconsistent. Several studies suggested a protective effect of statins use on the AMD risk [59]–[66] while many others reported either no protective effect [26], [40], [67]–[75] or even further a potential deleterious role [76], [77]. Finally, recent reviews reported that available data on RCT or prospective studies are insufficient to conclude that statins exhibit any role in preventing or delaying the onset or progression of AMD [78], [79].

The reasons underlying these inconsistencies are not clearly understood. A difficulty in the interpretation of the positive relationship between elevated HDL and AMD is that plasma lipids concentrations may not reflect tissue-specific effects. Indeed, results obtained with multiple biochemical, histochemical, and ultrastructural methods, mainly performed by Cristina Curcio’s team, suggest that RPE secretes apolipoprotein B (ApoB)-lipoprotein particles of unusual composition into BrM, where they accumulate with age eventually forming a lipid wall that is a precursor of basal linear deposit [9], [24]. In addition, an accumulation of oxidized ApoB100 lipoproteins in BrM, drusen and basal deposits have been observed in AMD. In atherosclerosis, the oxidation of ApoB100 lipoproteins lead to mainly innate immune system-mediated inflammation which initiates a cascade of pathological events ending with the formation of atherosclerotic plaques [24]. Thus, the oxidized ApoB100 in BrM have been suggested to initiate inflammation, innate immune response and drusen formation sharing with the “response to retention” hypothesis of atherosclerosis [24]. In this hypothesis, the retention of cholesterol-rich, atherogenic lipoproteins provokes a cascade of responses that lead to disease in a previously non-lesional artery. Similarly, it has been suggested that in AMD, the retention of a sub-endothelial apolipoprotein B may lead to the formation of AMD lesion [24]. However, this theory does not exclude the potential contribution of lipoprotein synthesized in the liver or in intestine transported by bloodstream [10].

The increasing number of studies associating high HDL with increased risk for AMD suggests the possibility of a real relationship between high HDL and AMD, which might be due to a dysfunction of HDL. Recent findings on strategies to reduce cardiovascular risk turned attention to HDL quality (the HDL functional capability such as anti-oxidative, anti-inflammatory, anti-apoptotic, anti-infectious or anti-thrombotic functions of HDL) rather than quantity. Some studies suggested that plasma HDL concentrations do not predict functionality and composition of HDL [80] and may be a potential factor of conflicting results in the literature [81]. Indeed, HDL are highly heterogeneous in structure (density, size, charge and protein) and biologic function [80]. The anti-oxidant and anti-inflammatory activities of HDL can become ineffective due to inflammation and other factors such as myeloperoxidase-mediated oxidation. Consequently, HDL may turn into dysfunctional, pro-inflammatory and pro-oxidant particles that promote LDL oxidation and impair cholesterol efflux and reverse cholesterol transport [80], [81]. Thus, recent studies suggested that testing functionality, composition (such as concentration of HDL subfractions) and anti-inflammatory properties of HDL will be better markers than testing plasma HDL concentration for identifying subjects at risk for coronary heart disease [81], [82]. Briefly, HDL subclasses can be classified by their density (HDL2, HDL3), their size (2a, 2b, 3a, 3b, 3c) their charge (pre-β, α, pre-α) and their main apolipoprotein content (apoA-I or both apoA1 and apoA-II) [81]. Under dyslipidaemic conditions, changes in HDL subfraction levels and functions are currently observed. HDL2 and more particularly HDL2b seem to be more predictive of coronary heart disease risk than HDL or HDL3 [81]. Accordingly, it has been reported that, HDL2b levels are lower in subjects with coronary artery disease compared to healthy subjects and inversely related to disease severity and progression of coronary lesions. Furthermore, the concentration of pre-β-particles has been found to increase in subjects with coronary artery and heart diseases and with myocardial infarction. Inversely, the levels of large α1- and pre-α particles have been reported to decrease in subjects with coronary heart disease in comparison with healthy subjects [81]. To our knowledge, no such studies have been conducted in the field of AMD.

One strength of the present study is that major potential confounding factors were taken into account, including socio-demographic status, factors related to vascular diseases, use of lipid-lowering medications and the major genetic polymorphisms. Indeed, regarding genetic polymorphism, GWAS studies of AMD identified new loci that were associated with blood lipids and particularly HDL levels [83], [84]. Most of the variants discovered affected blood lipid levels and are also associated with coronary artery disease [84]. In the present study, the association of AMD with HDL remained significant after taking these polymorphisms into account and no interactions of HDL concentration with genetic polymorphisms were identified.

One limitation of our study could come from the representativeness of the sample. The Alienor subsample tends to over-represent younger subjects and high socioeconomic status, among subjects participating to the 3C Study [49]. The individuals included in this study may accordingly be healthier and present different lifestyles, mainly concerning their diet and physical activity, in comparison with the general population. The distribution of vascular characteristics or the prevalence of eye diseases may have been affected due to these differences. However, participants from the 3C Study which were included in the Alienor study were not different from those who were not included for most parameters of interest in our study [49]. Moreover, as described previously [49], the age- and gender-specific prevalence rates of AMD in the Alienor study were similar to those observed in other studies performed in Europe [2], [85] and other industrialized countries [3]. Data collection was performed in the same way in all individuals irrespective of their AMD stage and photograph graders had no access to data related to plasma lipids, or any other cardiovascular or genetic characteristics. Consequently, we can assume that the error was not differential and was unlikely to have biased the estimation of any of the associations of AMD with vascular parameters.

Another limitation of our study is the relatively small number of late AMD cases, which may have induced insufficient statistical power for detecting some associations with serum lipids concentrations (in particular for HDL, which presented a tendency to an increased risk for late AMD, but did not reach statistical significance).

Lastly, a potential limitation is the high number of comparisons performed. Therefore, we cannot exclude that some of the observed associations were due to chance finding. However, we adjusted for this by using Bonferroni correction with many associations remaining highly significant. In addition our findings are mostly consistent with previous studies in this field.

In conclusion, our results suggest that elderly patients with high HDL concentration may be at increased risk for AMD. This association might reflect HDL dysfunction in AMD. In accordance with the field of cardiovascular diseases, epidemiological studies are needed on the associations of AMD with HDL subfractions and functions, in order to better understand the potential role of lipid metabolism, and in particular of cholesterol reverse transport, in AMD.

## Supporting Information

File S1 **Tables.** Table S1, Plasma lipid levels and statin and fibrate drug use according to baseline demographic, behavioural and anthropometric characteristics, in subjects of the Alienor study (N = 825); Table S2, Plasma lipid levels and statin and fibrate drug use according to baseline medical characteristics, in subjects of the Alienor study (N = 825); Table S3, Plasma lipid levels according to statin and fibrate drug use, in subjects of the Alienor study (N = 825).
(DOCX)
