# Supplementary material for: Elevated High-Density Lipoprotein Cholesterol and Age-Related Macular Degeneration: The Alienor Study
Source: PLoS One. 2014 Mar 7;9(3):e90973. doi: 10.1371/journal.pone.0090973 (PMC3946623; doi:10.1371/journal.pone.0090973)
Supplement: File S1 — Tables. Table S1, Plasma lipid levels and statin and fibrate drug use according to baseline demographic, behavioural and anthropometric characteristics, in subjects of the Alienor study (N = 825); Table S2, Plasma lipid levels and statin and fibrate drug use according to baseline medical characteristics, in subjects of the Alienor study (N = 825); Table S3, Plasma lipid levels according to statin and fibrate drug use, in subjects of the Alienor study (N = 825). (DOCX) [file pone.0090973.s001.docx]

**Supporting InformationTable S1. Plasma lipid levels and statin and fibrate drug use according to baseline demographic, behavioural and anthropometric characteristics, in subjects of the Alienor study (N=825)**

|  | **TC (mM)** | **LDL (mM)** | **HDL (mM)** | **TG (mM)** | **Statin*** | | | **Fibrate**^†^ | | |
| --- | --- | --- | --- | --- | --- | --- | --- | --- | --- | --- |
|  |  |  |  |  | **No (N=507)** | **Yes (N=318)** | | **No (N=663)** | | **Yes (N=162)** |
|  | **Mean (SD)** | **Mean (SD)** | **Mean (SD)** | **Mean (SD)** | **N (%)** | **N (%)** | | **N (%)** | | **N (%)** |
| **Age**^‡^**, years** |  |  |  |  |  |  | |  | |  |
| <80 (N=771) | 5.79 (0.97) | 3.64 (0.85) | 1.59 (0.39) | 1.22 (0.57) | 470 (92.7) | 301 (94.6) | | 618 (93.2) | | 153 (94.4) |
| ≥80 (N=54) | 5.77 (1.01) | 3.55 (0.86) | 1.66 (0.38) | 1.24 (0.64) | 37 (7.3) | 17 (5.3) | | 45 (6.8) | | 9 (5.6) |
| *P value*^§^ | *0.87* | *0.42* | *0.23* | *0.16* | *0.27* | | | *0.60* | | |
| **Sex** |  |  |  |  |  |  | |  | |  |
| Male (N=311) | 5.55 (0.91) | 3.55 (0.78) | 1.42 (0.33) | 1.28 (0.60) | 174 (34.3) | 137 (43.1) | | 260 (39.2) | | 51 (31.5) |
| Female (N=514) | 5.93 (0.99) | 3.69 (0.88) | 1.70 (0.39) | 1.18 (0.55) | 333 (65.7) | 181 (56.9) | | 403 (60.8) | | 111 (68.5) |
| *P value*^§^ | *<0.0001* | *0.01* | *P<0.0001* | *0.02* | *0.01* | | | *0.07* | | |
| **Educational level** |  |  |  |  |  |  | |  | |  |
| No education or Primary School (N=246) | 5.77 (0.95) | 3.65 (0.84) | 1.58 (0.37) | 1.19 (0.54) | 152 (30.0) | 94 (29.6) | | 190 (28.7) | | 56 (34.6) |
| Secondary School (N=221) | 5.75 (1.03) | 3.60 (0.90) | 1.58 (0.38) | 1.25 (0.56) | 121 (23.9) | 100 (31.4) | | 177 (26.7) | | 44 (27.2) |
| High School (N=88) | 5.81 (1.02) | 3.60 (0.91) | 1.71 (0.42) | 1.10 (0.50) | 61 (12.0) | 27 (8.5) | | 74 (11.2) | | 14 (8.6) |
| University (N=270) | 5.83 (0.94) | 3.67 (0.79) | 1.58 (0.41) | 1.26 (0.63) | 173 (34.1) | 97 (30.5) | | 222 (33.5) | | 48 (29.6) |
| *P value*^§^ | *0.84* | *0.75* | *0.04* | *0.08* | *0.06* | | | *0.41* | | |
| **Smoking** |  |  |  |  | (N=501) | | (N=316) | (N=656) | (N=161) | |
| Never (N=528) | 5.87 (0.96) | 3.69 (0.85) | 1.64 (0.40) | 1.19 (0.56) | 327 (65.3) | | 201 (63.6) | 424 (64.6) | 104 (64.6) | |
| [1-20[ pack-years (N=149) | 5.61 (1.02) | 3.56 (0.89) | 1.52 (0.36) | 1.67 (0.52) | 97 (19.4) | | 52 (16.5) | 116 (17.7) | 33 (20.5) | |
| ≥ 20 pack-years (N140) | 5.69 (0.97) | 3.55 (0.79) | 1.51 (0.40) | 1.38 (0.63) | 77 (15.4) | | 63 (19.9) | 116 (17.7) | 24 (14.9) | |
| *P value*^§^ | *0.008* | *0.12* | *<0.0001* | *0.0007* | *0.19* | | | *0.56* | | |
| **Physical activity (N=704)** |  |  |  |  | (N=434) | | (N=270) | (N=573) | (N=131) | |
| None or low (N=450) | 5.78 (0.99) | 3.61 (0.86) | 1.60 (0.40) | 1.24 (0.56) | 272 (62.7) | | 178 (65.9) | 361 (63.0) | 89 (67.9) | |
| Medium (N=170) | 5.86 (0.97) | 3.70 (0.87) | 1.62 (0.40) | 1.16 (0.56) | 109 (25.1) | | 61 (22.6) | 138 (24.1) | 32 (24.5) | |
| High (N=84) | 5.64 (0.87) | 3.60 (0.72) | 1.50 (0.35) | 1.72 (0.54) | 53 (12.2) | | 31 (11.5) | 74 (12.9) | 10 (7.6) | |
| *P value*^§^ | *0.25* | *0.49* | *0.07* | *0.30* | *0.67* | | | *0.23* | | |
| **BMI**^ǁ^  **(N=823)** |  |  |  |  | (N=506) | | (N=315) | (N=662) | (N=161) | |
| <25 (N=309) | 5.93 (0.97) | 3.71 (0.86) | 1.73 (0.41) | 1.09 (0.48) | 204 (40.3) | | 105 (33.1) | 254 (38.4) | 55 (34.2) | |
| [25-30[ (N=388) | 5.72 (0.97) | 3.62 (0.84) | 1.53 (0.36) | 1.25 (0.55) | 221 (43.7) | | 167 (52.7) | 308 (46.5) | 80 (49.7) | |
| ≥30 (N=126) | 5.64 (0.96) | 3.55 (0.80) | 1.44 (0.36) | 1.43 (0.72) | 81 (16.0) | | 45 (14.2) | 100 (15.1) | 26 (16.1) | |
| *P value*^§^ | *0.003* | *0.17* | *<0.0001* | *<0.0001* | *0.04* | | | *0.61* | | |

Abbreviations: TC: Total cholesterol; TG: Triglycerides; BMI: Body mass index; * Use of statin at one examination or more; ^†^ Use of fibrate at one examination or more; ^‡^ Age at blood sampling; ^§^ ANOVA or Student t-tests were performed for means comparison; χ^2^ tests were performed for frequency comparison; Statistically significant p-value <0.0083 (Bonferroni corrected, P=0.05/6=0.0083); ^ǁ^ calculated as weight in kilograms divided by height in meters squared;

**Table S2. Plasma lipid levels and statin and fibrate drug use according to baseline medical characteristics, in subjects of the Alienor study (N=825)**

|  | **TC (mM)** | **LDL (mM)** | **HDL (mM)** | **TG (mM)** | **Statin**^*^ | | **Fibrates**^†^ | |
| --- | --- | --- | --- | --- | --- | --- | --- | --- |
|  |  |  |  |  | **No (N=507)** | **Yes (N=318)** | **No (N=663)** | **Yes (N=162)** |
|  | **Mean (SD)** | **Mean (SD)** | **Mean (SD)** | **Mean (SD)** | **N (%)** | **N (%)** | **N (%)** | **N (%)** |
| **Cardiovascular disease (N=818)** |  |  |  |  | (N=501) | (N=317) | (N=657) | (N=161) |
| No (N=730) | 5.83 (0.97) | 3.67 (0.84) | 1.61 (0.40) | 1.20 (0.56) | 476 (95.0) | 254 (80.1) | 581 (88.4) | 149 (92.5) |
| Yes (N=88) | 5.48 (1.00) | 3.38 (0.90) | 1.47 (0.36) | 1.39 (0.60) | 25 (5.0) | 63 (19.9) | 76 (11.6) | 12 (7.4) |
| *P value*^‡^ | *0.001* | *0.002* | *0.001* | *<0.003* | *<0.0001* | | *0.13* | |
| **Hypertension**^§^ |  |  |  |  |  |  |  |  |
| No (N=204) | 5.85 (0.92) | 3.69 (0.83) | 1.66 (0.37) | 1.10 (0.45) | 144 (28.4) | 60 (18.9) | 167 (25.2) | 37 (22.8) |
| Yes (N=621) | 5.77 (0.99) | 3.62 (0.85) | 1.57 (0.40) | 1.26 (0.60) | 363 (71.6) | 258 (81.1) | 496 (74.8) | 125 (77.2) |
| *P value*^‡^ | *0.27* | *0.30* | *0.006* | *<0.0001* | *0.002* | | *0.53* | |
| **Diabetes**^ǁ^ **(N=800)** |  |  |  |  | (N=490) | (N=310) | (N=643) | (N=157) |
| No (N=699) | 5.81 (0.98) | 3.65 (0.84) | 1.62 (0.40) | 1.18 (0.54) | 448 (91.4) | 251 (81.0) | 567 (88.2) | 132 (84.1) |
| Yes (N=101) | 5.59 (0.96) | 3.51 (0.87) | 1.40 (0.34) | 1.49 (0.71) | 42 (8.6) | 59 (19.0) | 76 (11.8) | 25 (15.9) |
| *P value*^‡^ | *0.03* | *0.11* | *<0.0001* | *<0.0001* | *<0.0001* | | *0.16* | |

Abbreviations: TC: Total cholesterol; TG: Triglycerides; ^*^ Use of statin at one examination or more; ^†^ Use of fibrate at one examination or more; ^‡^ ANOVA or Student t-tests were performed for means comparison; χ^2^ tests were performed for frequency comparison; Statistically significant p-value <0.017 (Bonferroni corrected, P=0.05/3=0.017); ^§^ Average systolic blood pressure ≥ 140 mmHg and/or average Diastolic blood pressure ≥ 90 mmHg and/or antihypertensive medication use; ^ǁ^ Fasting glycemia ≥ 6.1 mmol/L or nonfasting glycemia ≥11.0 mmol/l or antidiabetic medication;

**Table S3. Plasma lipid levels according to statin and fibrate drug use, in subjects of the Alienor study (N=825)**

|  | **TC (mM)** | **LDL (mM)** | **HDL (mM)** | **TG (mM)** |
| --- | --- | --- | --- | --- |
|  |  |  |  |  |
|  | **Mean (SD)** | **Mean (SD)** | **Mean (SD)** | **Mean (SD)** |
| **Statin use**^*^ |  |  |  |  |
| No (N=507) | 5.75 (0.93) | 3.62 (0.78) | 1.63 (0.40) | 1.10 (0.46) |
| Yes (N=318) | 5.84 (1.05) | 3.66 (0.94) | 1.54(0.37) | 1.41 (0.67) |
| *P value*^†^ | *0.19* | *0.0003* | *0.002* | *<0.0001* |
| **Fibrate use**^‡^ |  |  |  |  |
| No (N=663) | 5.81 (0.97) | 3.65 (0.84) | 1.60 (0.39) | 1.21 (0.54) |
| Yes (N=162) | 5.71 (0.97) | 3.58 (0.88) | 1.56 (0.40) | 1.24 (0.69) |
| *P value*^†^ | *0.27* | *0.34* | *0.27* | *0.60* |

Abbreviations: TC: Total cholesterol; TG: Triglycerides; ^*^ Use of statin at one examination or more; ^†^ Student t-tests were performed for means comparison; Statistically significant p-value <0.025 (Bonferroni corrected, P=0.05/2=0.025); **^‡^** Use of fibrate at one examination or more
